# Postoperative mobility is associated with risk of reoperation and increased mortality after hip fracture: a nationwide cohort study of 33,486 patients

**DOI:** 10.2340/17453674.2026.45552

**Published:** 2026-03-05

**Authors:** Simon Storgaard JENSEN, Per Hviid GUNDTOFT, Jan-Erik GJERTSEN, Alma B PEDERSEN

**Affiliations:** 1 Department of Clinical Epidemiology, Aarhus University Hospital, Aarhus, Denmark; 2 Department of Clinical Medicine, Aarhus University, Aarhus, Denmark; 3 Department of Orthopaedic Surgery, Traumatology, Aarhus University Hospital, Aarhus, Denmark; 4 Department of Orthopaedic Surgery, Haukeland University Hospital, Bergen, Norway; 5 Department of Clinical Medicine, University of Bergen, Norway

## Abstract

**Background and purpose:**

Postoperative mobilization may influence the outcome following hip fracture. We aimed to examine whether regaining pre-fracture basic mobility on discharge is associated with subsequent risk of reoperation and mortality.

**Methods:**

Using nationwide Danish registries, we identified 33,486 patients ≥ 65 years who underwent hip fracture surgery between January 2016 and November 2021. Pre-fracture and discharge mobility were assessed with the Cumulated Ambulation Score (CAS, score 0–6 where 6 is best ambulation). The exposure was regaining pre-fracture CAS at discharge. Adjusted hazard ratios (aHRs) with 95% confidence intervals (CIs) for reoperation and mortality up to 365 days were estimated using Cox regression, adjusting for age, sex, surgery year, length of stay, and comorbidities.

**Results:**

On discharge, 19,329 patients (65%) had not regained pre-fracture CAS. The 30-day reoperation risk was similar among patients who had not regained CAS (2.9%) and those who had (2.5%; aHR 1.10, CI 0.94–1.29). At 365 days, not regaining CAS was associated with a lower reoperation risk (7.1% vs 8.6%; aHR 0.98, CI 0.89–1.07). A CAS loss of 1–2 points was associated with an increased 30-day reoperation risk (aHR 1.20, CI 1.00–1.44). CAS decline was consistently associated with higher mortality at 30 days (aHR 2.07, CI 1.82–2.35) and 365 days (aHR 1.77, CI 1.66–1.89), with progressively higher rates at greater CAS decline.

**Conclusion:**

We found no consistent association between failure to regain pre-fracture mobility on discharge and reoperation, although patients with a 1–2 point CAS loss experienced a modestly higher 30-day reoperation risk. In contrast, failure to regain pre-fracture mobility was strongly associated with increased mortality up to 1 year after hip fracture.

Hip fractures are a major global health concern among older patients. Mortality rates are around 24% within 1 year in Scandinavia, and up to 10% of patients require reoperation [1,2]. Additionally, many patients experience complications and long-term functional decline, resulting in substantial healthcare costs and decreased quality of life [3,4]. Early mobilization is a central component of multidisciplinary hip fracture care, enhancing functional recovery and reducing complication rates [5,6]. Current European guidelines recommend mobilization within 12–36 hours of surgery [7,8]. Early mobilization and the regaining of pre-fracture basic mobility during hospitalization are potentially modifiable factors that have been linked to long-term survival, reduced readmission rates, and decreased infection risk [8-11].

Recent meta-analyses of lower extremity fractures suggest that mobilization is associated with improved functional outcomes, while cohort studies have linked impaired mobility to a higher risk of deep infection and overall reoperation [12-14]. Similar mechanisms plausibly link impaired mobility to reoperation after a hip fracture. Reduced mobilization may impair tissue perfusion and wound healing, while also contributing to muscle atrophy, joint stiffness, and altered load transmission, potentially predisposing to fixation failure, dislocation, and infection in older patients [14,15]. However, impaired mobility also likely reflects underlying patient- and fracture-related vulnerability, including frailty and comorbidity [16,17]. Consequently, it remains uncertain whether impaired mobility is a causal determinant of reoperation or merely a marker of underlying vulnerability.

To date, no population-based studies have examined whether regaining pre-fracture basic mobility before discharge is associated with reoperation risk after hip fracture surgery. We aimed to examine the association between a decline in mobility and reoperations within 365 days after surgery. Further, we assessed the association between mobility decline and mortality, including evaluation of a potential dose–response relationship.

## Methods

We conducted a population-based cohort study using data from Danish national medical registries. In Denmark, both initial hip fracture surgeries and subsequent reoperations are exclusively managed within public hospitals as part of a universal, tax-funded healthcare system [18]. This paper follows the STROBE guidelines for cohort studies [19].

### Data sources

We identified patients using the Danish Multidisciplinary Hip Fracture Registry (DMHFR), which has systematically collected data since 2004 on all patients aged ≥ 65 years undergoing primary hip fracture surgery. Reporting is mandatory for all Danish public hospitals via a reimbursement-based system, and the completeness of the DMHFR is considered very high, although a small number of patients may not be registered. The positive predictive value for recorded hip fracture events in the DMHFR is 100%. Registry audits indicate approximately 90% completeness of CAS registrations as a quality indicator, while descriptive variables such as residency status and body mass index have completeness of approximately 91% and 85%, respectively [20,21].

Data was linked at the individual level using the unique civil registration number assigned to all residents by the Danish Civil Registration System (CRS). The CRS enables accurate linkage across registries and provides continuously updated information on vital status, emigration, and immigration.

The Danish National Patient Registry (DNPR) contains data on all hospital contacts, including admission and discharge dates, diagnosis codes, and surgical procedures [22]. The DNPR was used to identify comorbidities, treatments during hospitalization, and surgical procedures.

The Danish Psychiatric Central Research Register (DPCRR) contains nationwide data on psychiatric hospital admissions and outpatient contacts [23]. The DPCRR was used to identify the presence and category of mental disorder comorbidity.

### Baseline mobility

Pre-fracture mobility was assessed using the Cumulated Ambulation Score (CAS), which evaluates independence in 3 basic functional activities: (i) getting in and out of bed, (ii) sit-to-stand-to-sit from a chair with armrests, and (iii) indoor walking. Each activity is scored from 0 to 2 points (0 = not able despite human assistance and verbal guidance; 1 = able with human assistance and/or verbal guidance; 2 = able to perform safely without assistance or guidance), yielding a total score ranging from 0 to 6 [24]. CAS is a validated and reliable measuring tool of basic mobility after hip fracture and has been widely used in both clinical practice and registry-based studies across several European countries [24,25]. Both pre-fracture and discharge CAS were obtained from the DMHFR, where CAS reporting is mandatory.

Change in CAS from pre-fracture status to discharge was categorized as regained mobility (discharge CAS = pre-fracture CAS) or not regained mobility (discharge CAS < pre-fracture CAS). The primary exposure was regaining pre-fracture CAS on discharge (yes/no), and the secondary exposure was the number of CAS points lost, analyzed as a continuous variable.

### Outcomes

Reoperation was defined as any secondary hip-related surgical intervention, including closed reduction of prosthesis dislocation, occurring within 30 and 365 days after the index hip fracture surgery. Reoperations were identified in the DNPR using NOMESCO surgical procedure codes, in accordance with the Danish Clinical Quality Program – National Clinical Registries, designed to benchmark hospital performance and support quality improvement initiatives [26] (see Supplementary Table S1).

Data on all-cause mortality at 30 and 365 days after surgery was obtained from the CRS and linked to the DMHFR.

### Covariates

Length of hospital stay was obtained from the DNPR. The following covariates were collected at the time of hip fracture admission:

- Age and sex retrieved from the CRS. Age was categorized as 65–74, 75–84, 85–94, and ≥ 95 years.
- Information on surgery year (categorized as 2016–2017, 2018–2019, and 2020–2021), surgical delay (in hours, categorized as 0–12, > 12–24, > 24–36, > 36–48, > 48), body mass index (BMI categorized as underweight < 19, normal 19–24.9, overweight 25–29.9, obese ≥ 30, or missing), fracture type (ICD-10 codes: S72.0 femoral neck, S72.1 pertrochanteric, S72.2 subtrochanteric), and surgical procedure (NFJ: osteosynthesis of the femur; or NFB: primary prosthetic replacement of the hip joint) obtained from the DMHFR.
- Information on comorbidities assessed using ICD-10 diagnosis codes from the DNPR and DPCRR. Based on a Danish hip fracture study, we defined 26 clinically relevant comorbidities with a prevalence greater than 1% [27]. Comorbidities were assessed over the 10 years preceding the index hip fracture surgery and categorized as: cardiovascular, dementia, hepatic/gastrointestinal, malignant, mental disorders (e.g., depression and anxiety), metabolic, musculoskeletal, neurological/alcohol, pulmonary, and renal/hematological (see Supplementary Table S2).
- Living situation (categorized as own home/cohabitation, own home/alone, nursing home, and other/missing) obtained by combining marital and cohabitation status from the CRS with data on housing conditions from the DMHFR.

### Inclusion and exclusion

We included all patients ≥ 65 years with a surgically treated first-time unilateral low-energy hip fracture recorded in the DMHFR between January 2016 and November 2021. Patients who died or underwent reoperation before hospital discharge were excluded, as these events precluded assessment of CAS on discharge. Patients with missing CAS data were not excluded but were analyzed separately and compared with those with complete CAS data.

### Statistics

Patient characteristics were summarized according to the primary exposure. The primary outcome was reoperation, and the secondary outcome was mortality. Follow-up began on the date of hospital discharge and continued up to 365 days after surgery or until reoperation, death, emigration, or 31 December 2022, whichever came first. The cumulative incidence of reoperation was estimated using the Aalen–Johansen method, treating death as a competing risk and stratifying by regained CAS. Similarly, the cumulative incidence of mortality was calculated, stratified by regaining CAS. Cause-specific Cox proportional hazards regression was used to estimate crude and adjusted hazard ratios (aHRs) with 95% confidence intervals (CIs) for the relative rate of reoperation among patients who were alive and event-free. Patients contributed risk time until reoperation, death, or end of follow-up. This approach was chosen to assess associations between mobility status and event rates, while absolute risks were described using cumulative incidence estimates. For mortality, Cox proportional hazards regression was used to estimate crude and adjusted HRs for 30-day and 365-day all-cause mortality.

Cox proportional hazards regression models were adjusted for potential confounders identified using directed acyclic graphs, based on known or suspected causal pathways linking patient characteristics, hospital care, and postoperative outcomes. Age, sex, and comorbidities may affect both pre-fracture mobility and the risk of reoperation or mortality, while surgery year and length of stay may capture temporal trends. This approach adjusts for key confounders when estimating the association between regaining CAS and outcomes. The proportional hazards assumption was evaluated using log(–log) curves. The curves within each compared exposure group were approximately parallel without notable systematic divergence or crossover, indicating that the assumption was fulfilled. No time-dependent effects requiring model modification were identified.

In addition, we compared the complete-case cohort with patients who had missing CAS data to evaluate differences in patient characteristics and reoperation risk, thereby assessing the potential for selection bias. To investigate potential differences in the distribution of reoperations, we conducted an additional analysis restricted to major reoperations. Under this redefinition, NFH20 (closed reduction of a dislocated prosthesis) was excluded, as was NFU49 (removal of osteosynthesis material) when performed more than 6 months after the index surgery and recorded alongside T84.1 (mechanical complication of an internal fixation device), as such cases were considered to result from hardware irritation (e.g., screws or plates).

To explore potential effect modification, we additionally stratified by age, sex, and living situation to evaluate the association between early mobilizations and reoperation.

We assessed potential ceiling and floor effects in CAS scoring, recognizing that the possible score reduction is constrained by the pre-fracture CAS (e.g., a pre-fracture score of 2 permits a maximum decline of 2 points). Accordingly, analyses were stratified by pre-fracture CAS.

Analyses were conducted using R software (version 4.3.2; R Foundation for Statistical Computing, Vienna, Austria).

### Ethics, data sharing, use of AI, funding, and disclosures

The study was reported to the Danish Data Protection Agency through Aarhus University (Record number AU-2016-051-000001, sequential number 880). The data analyzed in this study was made available in anonymized form on Statistics Denmark’s servers. To protect the privacy of patients, individual-level data is not publicly disclosed, but access may be granted by the relevant authorities in Denmark. AI-based tools were used solely to assist with grammatical and punctuation improvements. The authors received no external funding and declare no conflicts of interest. Complete disclosure of interest forms according to ICMJE are available on the article page, doi: 10.2340/17453674.2026.45552

## Results

**Figure 1:**
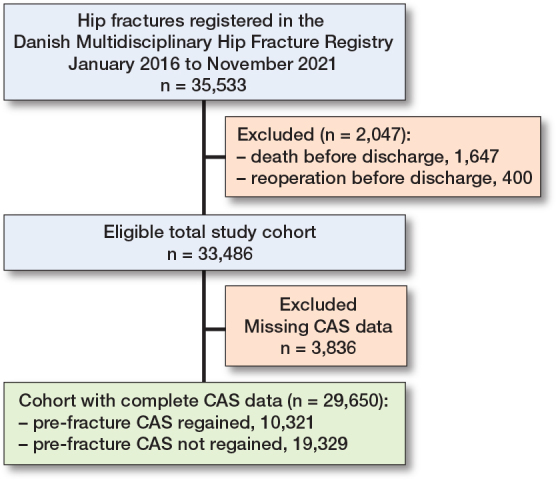
Flow diagram of the study population. CAS: Cumulated Ambulation Score.

33,486 patients were identified in the DMHFR as being discharged alive and without having undergone reoperation during the initial hospital stay. Of these, 29,650 (89%) had complete data on both pre-fracture and discharge CASs (Figure 1). 10,321 patients (35%) had regained their pre-fracture CAS, with a mean length of stay of 6.9 days (CI 6.8–7.0), while 19,329 (65%) had not regained their pre-fracture CAS, with a mean length of stay of 7.9 days (CI 7.8–8.0) (Table 1). Patients who regained their pre-fracture CAS were slightly younger, more often had femoral neck fractures treated with arthroplasty, were more frequently married, more commonly lived in their own homes, and had a lower comorbidity burden than those who did not regain their CAS (Table 1).

**Table 1:** Patient characteristics by regained Cumulated Ambulation Score (CAS) status. Values are count (%)unless otherwise specified

	Pre-fracture CAS regained		
	Yes	No	Total
Total	10,321 (100)	19,329 (100)	29,650 (100)
Female	7,015 (68)	13,223 (68)	20,238 (68)
Male	3,306 (32)	6,106 (32)	9,412 (32)
Age, years			
65–74	3,696 (36)	3,158 (16)	6,854 (23)
75–84	4,152 (40)	7,139 (37)	11,291 (38)
85–94	2,254 (22)	7,773 (40)	10,027 (34)
≥ 95	219 (2.1)	1,259 (6.5)	1,478 (5.0)
Surgery year			
2016–2017	3,711 (36)	6,268 (32)	9,979 (34)
2018–2019	3,348 (32)	6,285 (33)	9,633 (32)
2020–2021	3,262 (32)	6,776 (35)	10,038 (34)
Fracture type			
Femoral neck	6,855 (66)	10,121 (53)	16,976 (57)
Pertrochanteric	2,961 (29)	7,799 (40)	10,760 (36)
Subtrochanteric	505 (4.9)	1,409 (7.3)	1,914 (6.5)
Surgery type			
Osteosynthesis	6,068 (59)	12,616 (65)	18,684 (63)
Arthroplasty	4,253 (41)	6,713 (35)	10,966 (37)
Surgery delay, hours			
< 12	2,663 (26)	5,088 (26)	7,751 (26)
12 to < 24	4,765 (46)	8,730 (45)	13,495 (46)
24 to < 36	1,817 (18)	3,295 (17)	5,112 (17)
36 to < 48	630 (6.1)	1,279 (6.6)	1,909 (6.4)
≥ 48	446 (4.3)	937 (4.9)	1,383 (4.7)
Length of stay			
mean, days (CI)	6.9 (6.8–7.0)	7.9 (7.8–8.0)	7.6 (7.5–7.6)
Body mass index category			
< 19	836 (8.1)	1,689 (8.7)	2,525 (8.5)
19 to < 25	4,611 (45)	8,491 (44)	13,102 (44)
25 to < 30	2,608 (25)	4,673 (24)	7,281 (24)
≥ 30	828 (8.0)	1,728 (8.9)	2,556 (8.6)
Missing	1,438 (14)	2,748 (14)	4,186 (14)
Living situation			
Own home			
cohabitation	4,204 (41)	4,755 (25)	8,959 (30)
alone	4,049 (39)	7,145 (37)	11,194 (38)
Nursing home	1,141 (11)	5,876 (30)	7,017 (24)
Other/missing	927 (9.0)	1,553 (8.0)	2,480 (8.4)
Comorbidity clusters			
Cardiovascular	5,170 (50)	11,793 (61)	16,963 (57)
Dementia	361 (3.5)	1,953 (10)	2,314 (7.8)
Hepatic/gastrointestinal	514 (5.0)	1,098 (5.7)	1,612 (5.4)
Malignant	2,034 (20)	3,838 (20)	5,872 (20)
Mental disorders	466 (4.5)	1,175 (6.1)	1,641 (5.5)
Metabolic	2,157 (21)	4,855 (25)	7,012 (24)
Musculoskeletal	553 (5.4)	1,010 (5.2)	1,563 (5.3)
Neurological/alcohol	880 (8.5)	2,262 (12)	3,142 (11)
Pulmonary	1,377 (13)	3,007 (16)	4,384 (15)
Renal/hematological	1,933 (19)	5,422 (28)	7,355 (25)

Comorbidities are measured by prevalence within 10 years before hip fracture surgery.

IQR: interquartile range.

Patients not included due to missing data on pre-fracture and/or discharge CAS (n = 3,836) were more often men, had a longer surgical delay and hospital stay, and had more missing information on BMI and living situation (see Supplementary Table S3), but did not differ in age or comorbidities compared with patients with complete data.

### Reoperations

At 30 days, there was a slightly higher crude cumulative incidence of reoperation among patients who did not regain their pre-fracture CAS compared with those who did (2.9% vs 2.5%), corresponding to an HR of 1.18 (CI 1.02–1.37). After adjustment, there was no significant association with an aHR of 1.10 (CI 0.94–1.29) (Table 2).

**Table 2:** Reoperation and mortality by regained Cumulated Ambulation Score (CAS) status

Factor Follow-up	CAS regained	Cumulative incidence **^a^** % (CI)	Crude HR (CI)	Adjusted HR **^b^** (CI)
Reoperation				
30 days	Yes	2.5 (2.2–2.8)	Ref.	Ref.
	No	2.9 (2.6–3.1)	1.2 (1.02–1.4)	1.1 (0.94–1.3)
365 days	Yes	8.6 (8.1–9.2)	Ref.	Ref.
	No	7.1 (6.7–7.4)	0.91 (0.83–0.99)	0.98 (0.89–1.1)
Mortality				
30 days	Yes	2.8 (2.5–3.1)	Ref.	Ref.
	No	8.9 (8.5–9.3)	3.3 (2.9–3.8)	2.1 (1.8–2.4)
365 days	Yes	12 (12–13)	Ref.	Ref.
	No	29 (27–30)	2.6 (2.4–2.7)	1.8 (1.7–1.9)

a Cumulative incidence for reoperation, treating death as a competing risk.

b Adjusted by age, sex, surgery year, length of hospital stay, and comorbidities.

Abbreviations: CAS: Cumulated Ambulation Score, CI: 95% confidence interval, HR: hazard ratio, Ref.: reference.

**Figure 2:**
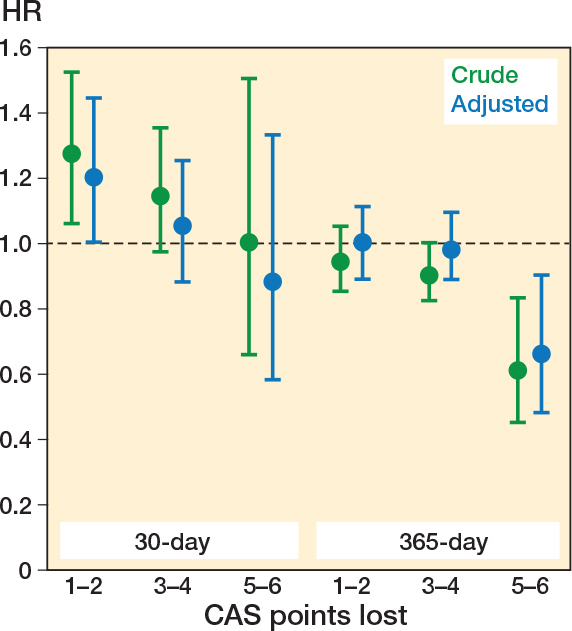
Reoperation rates by decline in Cumulated Ambulation Score (CAS) after hip fracture surgery, adjusted by age, sex, year of surgery, length of hospital stay, and comorbidity clusters. HR: hazard ratio with 1 (0 CAS points lost) as reference.

When analyzed according to the number of CAS points lost on discharge, patients with a loss of 1–2 points in CAS had an increased risk of reoperation within 30 days compared with those who had regained mobility (aHR 1.20, CI 1.00–1.44). Patients with a loss of 3–4 points and 5–6 points in CAS had a similar risk of reoperation within 30 days to those who had regained their pre-fracture mobility on discharge (aHR 1.05, CI 0.88–1.33 and aHR 0.88, CI 0.58–1.33, respectively) (Figure 2).

Among patients who regained their pre-fracture mobility, the 365-day cumulative incidence of reoperation was 8.6% (CI 8.1–9.2), compared with 7.1% (CI 6.7–7.4) among those who did not (aHR 0.98, CI 0.89–1.07) (see Table 2). When analyzed according to the number of CAS points lost on discharge, patients with a loss of 1–2 points and 3–4 points did not have an increased reoperation rate compared with those who regained their pre-fracture mobility (aHR 1.00, CI 0.89–1.11, and aHR 0.98, CI 0.89–1.09, respectively). Patients with a loss of 5–6 points had a significantly lower rate of reoperation (aHR 0.66, CI 0.48–0.90) (see Figure 2).

Patients with missing CAS data had a higher risk of reoperation than those with complete CAS information at 30 days, but no difference was observed at 365 days (Supplementary Table S4).

When restricting the outcome to major reoperations, the cumulative incidences decreased as expected. However, the crude and adjusted hazard ratios showed minimal to no differences compared with the primary analysis (see Supplementary Tables S5 and S6).

Stratified analyses showed that the association between decline in CAS and reoperation was largely consistent across age, sex, and living arrangements at both 30 and 365 days. Across strata, the highest reoperation rates were observed among patients with a 1–2-point loss in CAS, with only minor deviations.

Patients with a pre-fracture CAS of 5 or 6 who experienced a 1–2-point loss had the highest reoperation rates at both 30 and 365 days of follow-up, indicating a floor effect consistent with the main findings (Supplementary Figures S1 and S2).

### Mortality

**Figure 3:**
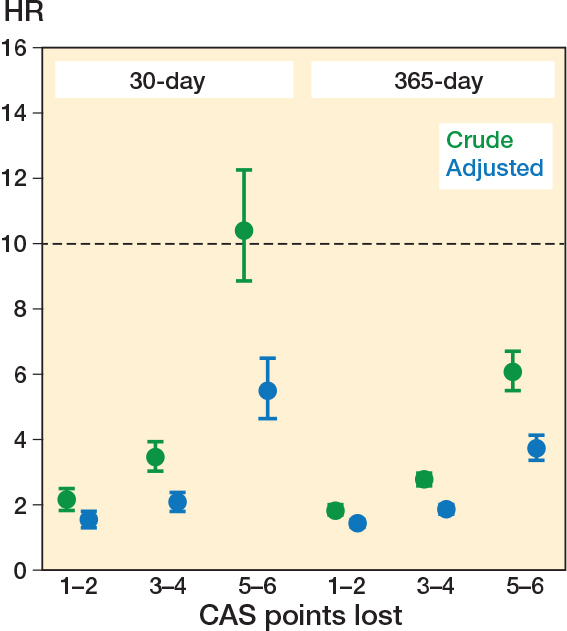
Mortality rates by decline in Cumulated Ambulation Score (CAS) after hip fracture surgery, adjusted by age, sex, year of surgery, length of hospital stay, and comorbidity clusters. HR: hazard ratio with 1 (0 CAS points lost) as reference.

Among patients who regained their pre-fracture CAS, the 30-day cumulative mortality was 2.8% (CI 2.5–3.1), compared with 8.9% (CI 8.5–9.3) among those who did not. This corresponded to an aHR of 2.07 (CI 1.82–2.35) (see Table 2). When analyzed according to the number of CAS points lost on discharge, all categories of functional decline were associated with significantly increased 30-day mortality compared with patients who regained their pre-fracture mobility. The aHR was 1.55 (CI 1.33–1.80) for patients with a loss of 1–2 points, 2.07 (CI 1.81–2.37) for a loss of 3–4 points, and 5.48 (CI 4.30–6.49) for a loss of 5–6 points in CAS (Figure 3).

Similar patterns were observed for 365-day mortality. Patients who did not regain their pre-fracture CAS had higher mortality (28.6% vs 12.4%), corresponding to an aHR of 1.77 (CI 1.66–1.89). Mortality at 365 days increased progressively with the extent of CAS decline at discharge (see Table 2, Figure 3).

Mortality was higher among patients with missing CAS data at both 30 and 365 days compared with those with complete CAS information. Overall, outcomes of patients with missing CAS data were comparable to those with a decline in CAS (see Supplementary Table S4).

## Discussion

This is the first study examining the association between changes in mobility status and reoperation risk after hip fracture. We aimed to examine whether regaining pre-fracture basic mobility on discharge is associated with subsequent reoperation risk and mortality. We found that a decline inf pre-fracture mobility on hospital discharge was not associated with any significant increase in overall reoperation risk. Patients with a moderate decline in CAS (1–2 points) had a modestly higher 30-day reoperation risk, but no clear association was observed at 365 days. In contrast, not regaining pre-fracture mobility was strongly associated with increased mortality at both 30 and 365 days, with risks rising progressively with the extent of CAS decline, demonstrating a clear dose–response relationship. These associations were robust across patient subgroups and after accounting for potential floor effects.

Earlier Danish register studies found an increased risk of any infection among patients with delayed mobilizations [8], and higher infection risk with the degree of decline in CAS [10], respectively, and increased readmission rate for patients who did not regain their pre-fracture CAS on discharge [9].

Mobility is a strong predictor of short- and long-term mortality following hip fracture [9,11]. We extend this evidence by showing a dose–response relationship between mortality and the degree of decline in CAS.

The inverse association between reoperation and mortality suggests that competing risks and treatment preferences influence reoperation decisions. In very frail patients with limited life expectancy, reoperation may be avoided following shared decision-making, even in the presence of surgical complications. Prior literature on primary hip fracture surgery shows that nonoperative management is sometimes chosen on medical and preference-based grounds and is associated with high short-term mortality in very frail patients [28,29]. These considerations are likely to extend to reoperation, reflecting both surgical need and clinical decision-making shaped by frailty, comorbidity, and competing mortality risk.

Our study highlights the complex interplay between mobility recovery, reoperation risk, and mortality following a hip fracture. Among patients with extensive multimorbidity and limited life expectancy, clinicians may refrain from recommending reoperation unless the indication is strong (e.g., infection or periprosthetic fracture). Reoperations may thus reflect not only clinical need but also cautious treatment strategies for patients with frailty. Conversely, reduced routine postoperative follow-up in many healthcare settings may disproportionately affect multimorbid and immobile patients, increasing the risk of under-recognized complications and missed opportunities for timely reoperation.

Our findings emphasize that mobilization and physical therapy remain crucial, as any decline in CAS is associated with substantially higher mortality. An increased risk of reoperation was observed only among patients with pre-fracture CAS 5–6 who experienced a moderate decline in mobility. In patients with high pre-fracture functional capacity, a ceiling effect of CAS may mask differences in functional reserve. In this group, even small postoperative reductions in function may exceed what is captured by this simple measure of baseline mobility and may represent a clinically meaningful signal for closer postoperative assessment and surgical follow-up.

### Strengths

A major strength of this study is its large, nationwide design, based on routinely and prospectively collected individual-level data from several high-quality Danish registries. Hip fracture surgeries recorded in the DMHFR have near-complete coverage, with procedure codes for primary operations showing a positive predictive value of 100% [21]. The validity of reoperation registration is generally high, although completeness may be somewhat lower than for primary procedures, as reported in other Scandinavian registry studies [30].

The use of the CAS is a key strength, as CAS is a internationally widely used measure of basic mobility in both clinical practice and registry-based research. CAS represents a potentially modifiable outcome [25]. Pre-fracture CAS is based on patient reports and, when necessary, supplemented by relatives or caregivers. This limitation may introduce some degree of misclassification. However, CAS has demonstrated good reliability in this setting, and any misclassification is likely to be non-differential [31]. Missing CAS data is another potential limitation. To minimize bias, patients with missing CAS were not excluded and were analyzed as a separate category, ensuring that all available information contributed to the study findings.

### Limitations

Length of hospital stay and postoperative care may influence mobility and subsequent outcomes after a hip fracture. Shorter hospital stays have been associated with increased short-term mortality, whereas geriatric or orthogeriatric care pathways and team-based rehabilitation have been linked to lower mortality and improved functional recovery [32,33]. However, the length of stay reflects heterogeneous clinical trajectories, with length of stay being short among severely ill patients with limited recovery potential, nursing home patients, and those who recover rapidly. Prolonged stays may allow additional time for mobilizations but may also indicate postoperative complications. In this context, a decline in CAS on discharge may reflect a combination of pre-existing vulnerability, frailty, surgical factors, and postoperative events, including differences in care pathways, rehabilitation intensity, and in-hospital mobilizations. It is also important to recognize that a suboptimal primary surgery may itself cause pain or functional limitation, contributing to a decline in CAS and increasing the likelihood of subsequent reoperation [33].

We observed an inverse association between reoperation and mortality, with patients experiencing greater functional decline showing a lower risk of reoperation. Mortality likely contributes to this pattern as a competing event. To account for this, we used the Aalen–Johansen estimator, which appropriately handles death as a competing risk and avoids the overestimation associated with Kaplan–Meier methods. Although nearly all patients with hip fractures in Denmark undergo primary surgery, selection bias remains a potential concern regarding reoperation.

### Conclusion

A decline in CAS was not consistently associated with an increased risk of reoperation, although patients with a 1–2 point decline experienced a modestly higher 30-day reoperation risk. In contrast, larger declines in CAS were consistently associated with progressively higher short- and medium-term mortality, demonstrating a clear dose–response relationship. The limited association between impaired mobility and reoperation may reflect competing mortality risk and clinical decision-making in frail patients rather than the absence of postoperative complications.

*In perspective*, these findings underscore that postoperative mobility decline on discharge is a strong prognostic indicator of mortality.

### Supplementary data

Tables S1–S6 and Figures S1–S2 are available as Supplementary data on the article page, doi: 10.2340/17453674.2026.45552

## Supplementary Material



## Notes

SSJ, PHG, JEG, and ABP: conceived and designed the study. SSJ and ABP: contributed to the statistical analysis, accessed and verified the data. SSJ and ABP: wrote the paper. SSJ, PHG, JEG, and ABP: revised the paper critically for important intellectual content. Final approval and agreement of accountability for all aspects of the work: SSJ, PHG, JEG, and ABP.

Handling co-editors: Cecilia Rogmark and Jonas Ranstam

*Acta* thanks Bård Erik Bogen and Sebastian Mukka for help with peer review of this manuscript.
