# Supplementary material for: Postoperative mobility is associated with risk of reoperation and increased mortality after hip fracture: a nationwide cohort study of 33,486 patients
Source: Acta Orthop. 2026 Mar 5;97:156–63. doi: 10.2340/17453674.2026.45552 (PMC12964105; doi:10.2340/17453674.2026.45552)
Supplement: Supplementary file 1 [file ActaO-97-45552-s1.pdf]

Online Supplementary Material

Journal

Acta Orthopaedica

Article title

Postoperative decline in pre-fracture mobility is associated with risk of reoperation and mortality after hip fracture: a nationwide cohort study of 33,486 patients

Authors and affiliations

Simon Storgaard Jensen[1,2], Per Hviid Gundtoft[3], Jan-Erik Gjertsen[4,5], Alma B. Pedersen[1,2]

[1]Department of Clinical Epidemiology, Aarhus University Hospital, Aarhus, Denmark.
[2]Department of Clinical Medicine, Aarhus University, Aarhus, Denmark.
[3]Department of Orthopedic Surgery, Traumatology, Aarhus University Hospital, Aarhus, Denmark.
[4]Department of Orthopedic Surgery, Haukeland University Hospital, Bergen, Norway.
[5]Department of Clinical Medicine, University of Bergen, Norway.

Corresponding Author

Simon Storgaard Jensen
ssj@clin.au.dk

Supplementary Table S1. – NOMESCO Classification of Surgical Procedures (NCSP) used for defining reoperations

Classification of reoperation from primary surgery	Examples and elaboration	NCSP Codes
**Fracture of head and neck of femur, S72.0 + Fracture surgery of femur, NFJ**		
NFB0-99	Primary prosthetic replacement of hip joint	NFB, NFB0, NFB01, NFB02, NFB03, NFB09, NFB1, NFB11, NFB12, NFB13, NFB19, NFB20, NFB30, NFB40, NFB59, NFB99
NFC0-99	Secondary prosthetic replacement of hip joint	NFC, NFC0, NFC01, NFC02, NFC03, NFC09, NFC1, NFC11, NFC12, NFC13, NFC19, NFC2, NFC20, NFC21, NFC22, NFC23, NFC29, NFC3, NFC30, NFC31, NFC32, NFC33, NFC39, NFC4, NFC40, NFC41, NFC42, NFC43, NFC49, NFC59, NFC99
NFW0-99	Reoperations on hip or thigh	NFW, NFW49, NFW59, NFW69, NFW79, NFW89, NFW99
NFG09	Excision arthroplasty of hip joint	NFG09
NFU0-99	Removal of implants and external fixation devices from hip and femur	NFU, NFU0, NFU01, NFU02, NFU09, NFU1, NFU10, NFU11, NFU12, NFU19, NFU39, NFU49, NFU89, NFU89A, NFU89B, NFU99
NFS0-99	Operations for infection of tendons, joints and bone of hip and thigh	NFS, NFS19, NFS29, NFS49, NFS59, NFS99
**Pertrochanteric fracture, S72.1 or Subtrochanteric fracture of femur, S72.2 + Fracture surgery of femur, NFJ**		
NFB0-99	Primary prosthetic replacement of hip joint	NFB, NFB0, NFB01, NFB02, NFB03, NFB09, NFB1, NFB11, NFB12, NFB13, NFB19, NFB20, NFB30, NFB40, NFB59, NFB99
NFC0-99	Secondary prosthetic replacement of hip joint	NFC, NFC0, NFC01, NFC02, NFC03, NFC09, NFC1, NFC11, NFC12, NFC13, NFC19, NFC2, NFC20, NFC21, NFC22, NFC23, NFC29, NFC3, NFC30, NFC31, NFC32, NFC33, NFC39, NFC4, NFC40, NFC41, NFC42, NFC43, NFC49, NFC59, NFC99
NFW0-99	Reoperations on hip or thigh	NFW, NFW49, NFW59, NFW69, NFW79, NFW89, NFW99
NFG09	Excision arthroplasty of hip joint	NFG09
NFU0-99	Removal of implants and external fixation devices from hip and femur	NFU, NFU0, NFU01, NFU02, NFU09, NFU1, NFU10, NFU11, NFU12, NFU19, NFU39, NFU49, NFU89, NFU89A, NFU89B, NFU99
NFS0-99	Operations for infection of tendons, joints and bone of hip and thigh	NFS, NFS19, NFS29, NFS49, NFS59, NFS99
**Fracture of head and neck of femur, S72.0 or Pertrochanteric fracture, S72.1 or Subtrochanteric fracture of femur, S72.2 + Primary prosthetic replacement of hip joint, NFB**		

NFB0-99	Primary prosthetic replacement of hip joint	NFB, NFB0, NFB01, NFB02, NFB03, NFB09, NFB1, NFB11, NFB12, NFB13, NFB19, NFB20, NFB30, NFB40, NFB59, NFB99
NFC0-99	Secondary prosthetic replacement of hip joint	NFC, NFC0, NFC01, NFC02, NFC03, NFC09, NFC1, NFC11, NFC12, NFC13, NFC19, NFC2, NFC20, NFC21, NFC22, NFC23, NFC29, NFC3, NFC30, NFC31, NFC32, NFC33, NFC39, NFC4, NFC40, NFC41, NFC42, NFC43, NFC49, NFC59, NFC99
NFW0-99	Reoperations on hip or thigh	NFW, NFW49, NFW59, NFW69, NFW79, NFW89, NFW99
NFG09	Excision arthroplasty of hip joint	NFG09
NFH0-29	Miscellaneous operations on hip joint	NFH, NFH0, NFH00, NFH01, NFH02, NFH2, NFH20, NFH21, NFH22, NFH29
NFU0-99	Removal of implants and external fixation devices from hip and femur	NFU, NFU0, NFU01, NFU02, NFU09, NFU1, NFU10, NFU11, NFU12, NFU19, NFU39, NFU49, NFU89, NFU89A, NFU89B, NFU99
NFS0-99	Operations for infection of tendons, joints and bone of hip and thigh	NFS, NFS19, NFS29, NFS49, NFS59, NFS99
**Infection (T84.5 or T84.6 or T84.7) + S72.0 or S72.1 or S72.2 + NFB or NJF**		
NFW69	Reoperation for deep infection in surgery of hip or thigh. For infection at the site of target structures	NFW69
NFS0-99	Operations for infection of tendons, joints and bone of hip and thigh	NFS, NFS19, NFS29, NFS49, NFS59, NFS99
NFU0-99	Removal of implants and external fixation devices from hip and femur	NFU, NFU0, NFU01, NFU02, NFU09, NFU1, NFU10, NFU11, NFU12, NFU19, NFU39, NFU49, NFU89, NFU89A, NFU89B, NFU99

Supplementary Table S2. – International Classification of Disease 10th Edition (ICD-10) codes used for defining comorbidities

Comorbidities/Category	Examples and elaboration on comorbidities	ICD-10 Codes
Cardiovascular		
Cerebrovascular disease	Ischemia and hemorrhagic stroke; Transient ischemic attack; Etc.	I60-I69; G45-G46
Heart arrhythmia	Atrioventricular block; Paroxysmal tachycardia; Atrial fibrillation and flutter; Etc.	I44.1 - I44.3; I45.6; I45.9; I47-I49; R00.0; R00.1; R00.8; T82.1; Z45.0; Z95.0
Heart failure	Hypertensive heart disease; Ischemic cardiomyopathy; Myocarditis; Chronic ischemic heart disease; Heart failure; Etc.	I11.0; I13.0; I13.2; I09.9; I25.5; I42.0; I42.5 - I43; I50; P29.0
Hypertension	Essential hypertension; Hypertensive heart disease or kidney disease; Secondary hypertension; Etc.	I10-I13; I15
Hypotension	Orthostatic hypotension; Etc.	I95; G90.9A
Myocardial infarction	Acute myocardial infarction; Complications to acute myocardial infarction; Etc.	I21-I23
Peripheral vascular disease	Atherosclerosis; Aortic or peripheral aneurysm and dissection; Arterial embolism and thrombosis; Claudication; Etc.	I70-I74; I77
Valvular heart disease	Mitral and aortic valve disease; Etc.	A52.0; I05-I08; I09.1; I09.8; I34-I39; Q23.0-Q23.3; Z95.2-Z95.4
Organic Mental Disorders (Dementia)		
Organic, including symptomatic, mental disorders (Dementia)	Includes dementia in Alzheimer's disease, vascular dementia, etc.	F00-F03, G30
Malignancy		
Any solid tumor	Malignant neoplasms including brain cancer, lung cancer, breast cancer, melanoma; Etc.	C00-C75
Hematologic cancer	Lymphoma; Malignant myeloma; Leukemia; Etc.	C81-C85; C88; C90-C96
Metastatic solid tumor	Malignant neoplasm with metastasis; Etc.	C76-C80
Metabolic		
Diabetes, complicated	Type I and II diabetes with kidney, ophthalmic, circulatory complication; Etc.	E10.2-E10.8; E11.2-E11.8
Diabetes, uncomplicated	Type I and II diabetes without complications	E10.0, E10.1; E10.9, E11.0; E11.1; E11.9
Hypercholesterolemia	Hypercholesterolemia; Etc.	E78.0
Thyroid disease	Iodine-deficiency; Non-toxic goiter; Thyrotoxicosis; Hypothyroidism; Etc.	E00-05; E06.2; E89.0
Mental Disorders		
Mental and behavioural disorders due to psychoactive substance use	Includes use of alcohol, cannabis, cocaine, nicotine, opioids, sedatives, hypnotics, anxiolytics, etc.	F10-F13; F19
Schizophrenia, schizotypal and delusional disorders	Includes schizophrenia, schizotypal disorders, schizoaffective disorders and other psychotic disorders.	F20-F29

Mood [affective] disorders	Manic disorders, bipolar disorders and major, moderate or mild Depression	F30; F31; F32.2; F32.3; F33.2; F33.3; F32.0; F32.1; F32.8; F32.9; F33.0; F33.1; F33.8; F33.9; F34; F38; F39
Neurotic, stress-related and somatoform disorders	Includes anxiety disorders, phobias, obsessive-compulsive disorders, etc.	F40-45; F48
Behavioral syndromes associated with physiological disturbances and physical factors	Includes anorexia nervosa, bulimia nervosa, etc.	F50.0; F50.2; F50.5; F50.9
Disorders of adult personality and behavior	Includes paranoid, schizoid, dissocial, emotionally unstable personality disorder, etc.	F60-F61
Mental retardation	Includes mild, moderate, severe mental retardation, etc.	F70-F79
Disorders of psychological development	Includes autism spectrum disorders	F82; F84
Behavioral and emotional disorders with onset usually occurring in childhood and adolescence	Includes attention-deficit hyperactivity disorder, conduct disorders, childhood emotional disorders, etc.	F90; F98.8; F98.9
Musculoskeletal		
Rheumatic disease	Sarcoidosis; Rheumatoid arthritis; Vasculitis; Systemic lupus erythematosus; Systemic sclerosis; Ankylosing spondylitis; Etc.	D86; L94.0; L94.1; L94.3; M05-M06; M08-M09; M12.0; M12.3; M30-M36; M45; M46.1; M46.8; M46.9
Neurological/Alcohol		
Alcohol overuse	Mental and behavioral disorders due to use of alcohol; Niacin deficiency; Alcoholic cardiomyopathy; Alcoholic liver disease; Etc.	F10; E52; G62.1; I42.6; K29.2; K70.0; K70.3; K70.9; T51; Z50.2; Z71.4; Z72.1
Neurological disorders	Huntington's disease; Parkinson's disease; Multiple sclerosis; Epilepsy; Encephalopathy; Etc.	G10-G13; G20-G22; G25.4; G25.5; G31.2; G31.8; G31.9; G32; G35-G37; G40-G41; G93.1; G93.4; R47.0; R56
Pulmonary		
Chronic pulmonary disease	Bronchitis; Emphysema; Chronic obstructive pulmonary disease; Asthma; Pneumoconiosis; Interstitial lung disease; Etc.	J40-J47; J60-J67; J68.4; J70.1; J70.3; J84.1; J92.0; J96.1; J98.2; J98.3
Pulmonary circulation disorders	Pulmonary embolism; Pulmonary hypertension; Etc.	I26; I27; I28.0; I28.8; I28.9
Renal/Hematological		
Anemia	Deficiency anemia; Blood loss anemia; Aplastic anemia; Etc.	D50-59; D60-64
Fluid and electrolyte disorders	Volume depletion; Disorders of fluid, electrolyte and acid-base balance; Etc.	E22.2; E86-E87
Renal disease	Hypertensive chronic kidney disease; Glomerular disease; Renal tubulointerstitial diseases; Kidney failure; Etc.	I12-I13; N00-N05; N07; N11; N14; N17-N19; Q61

**Supplementary Table S3. Patient characteristics of the total cohort**			
	**CAS status**		
	**CAS available**	**CAS missing**	**Total**
**Total**	29,650 (100)	3,836 (100)	33,486 (100)
Female	20,238 (68)	2,488 (65)	22,726 (68)
Male	9,412 (32)	1,348 (35)	10,760 (32)
**Age, years**			
65–74	6,854 (23)	924 (24)	7,778 (23)
75–84	11,291 (38)	1,459 (38)	12,750 (38)
85–94	10,027 (34)	1,251 (33)	11,278 (34)
≥95	1,478 (5.0)	202 (5.3)	1,680 (5.0)
**Surgery year**			
2016–2017	9,979 (34)	1,301 (34)	11,280 (34)
2018–2019	9,633 (32)	1,426 (37)	11,059 (33)
2020–2021	10,038 (34)	1,109 (29)	11,147 (33)
**Fracture type**			
Femoral neck	16,976 (57)	2,163 (57)	19,139 (57)
Pertrochanteric	10,760 (36)	1,393 (36)	12,153 (36)
Subtrochanteric	1,914 (6.5)	280 (7.3)	2,194 (6.6)
**Surgery type**			
Osteosynthesis	18,684 (63)	2,452 (64)	21,136 (63)
Arthroplasty	10,966 (37)	1,384 (36)	12,350 (37)
**Surgery delay, hours**			
<12	7,751 (26)	776 (20)	8,524 (26)
12 to <24	13,495 (46)	1,619 (42)	15,114 (45)
24 to <36	5,112 (17)	701 (19)	5,813 (17)
36 to <48	1,909 (6.4)	317 (8.3)	2,226 (6.7)
48	1,383 (4.7)	423 (11)	1,806 (5.4)
**Length of stay**			
Mean, days (CI)	7.6 (7.5–7.6)	9.4 (9.1–9.6)	7.8 (7.7–7.8)
**BMI category**			
<19	2,525 (8.5)	242 (6.3)	2,767 (8.3)
19 to <25	13,102 (44)	1,174 (31)	14,276 (43)
25 to <30	7,281 (24)	657 (17)	7,938 (24)
≥30	2,556 (8.6)	238 (6.2)	2,794 (8.3)
Missing	4,186 (14)	1,525 (40)	5,711 (17)
**Living situation**			
Own home, cohab.	8,959 (30)	806 (21)	9,765 (29)
Own home, alone.	11,194 (38)	1,010 (26)	12,204 (37)
Nursing home	7,017 (24)	820 (22)	7,837 (23)
Other/missing	2,480 (8.4)	1,200 (31)	3,680 (11)
**Comorbidity clusters**			
Cardiovascular	16,963 (57)	2,249 (59)	19,212 (57)
Dementia	2,314 (7.8)	363 (10)	2,677 (8.0)
Hepatic/gastrointestinal	1,612 (5.4)	222 (5.8)	1,834 (5.5)
Malignant	5,872 (20)	774 (20)	6,646 (20)
Mental disorders	1,641 (5.5)	277 (7.2)	1,918 (5.7)
Metabolic	7,012 (24)	966 (25)	7,978 (24)
Musculoskeletal	1,563 (5.3)	197 (5.1)	1,760 (5.3)
Neurological/alcohol	3,142 (11)	487 (13)	3,629 (11)
Pulmonary	4,384 (15)	599 (16)	4,983 (15)
Renal/hematological	7,355 (25)	1,080 (28)	8,435 (25)

Values are *n (%)* unless otherwise specified.
Comorbidities are measured by prevalence within 10 years before hip fracture surgery.
Abbreviations: CAS: Cumulated Ambulation Score, IQR: interquartile range. BMI: body mass index. Cohab.: cohabitation.

Supplementary Table S4. **Reoperation and mortality by regained CAS status.**

	Follow-up	CAS status	Cumulative incidence [a] % (CI)	Crude HR (CI)	Adjusted HR [b] (CI)
**Reoperation**	30 days	Available	2.7 (2.6–2.9)	Ref.	Ref.
		Missing	3.0 (2.5–3.6)	1.13 (0.93–1.37)	1.13 (0.93–1.37)
	365 days	Available	7.6 (7.3–7.9)	Ref.	Ref.
		Missing	7.3 (6.5–8.2)	1.01 (0.89–1.14)	1.01 (0.89–1.14)
**Mortality**	30 days	Available	6.8 (6.5–7.1)	Ref.	Ref.
		Missing	10.1 (9.1–11.0)	1.52 (1.36–1.69)	1.44 (1.29–1.61)
	365 days	Available	23.0 (22.5–23.4)	Ref.	Ref.
		Missing	28.8 (27.4–30.3)	1.32 (1.24–1.40)	1.28 (1.20–1.36)

[a] Cumulative incidence for reoperation, treating death as a competing risk.
[b] Adjusted by age, sex, year of surgery, length of hospital stay, and comorbidities.
Abbreviations: CAS: Cumulated Ambulation Score, CI: 95% confidence interval, HR: hazard ratio, Ref.: reference.

Supplementary Table S5. Major reoperations by change in CAS status.					
	**Follow-up**	**CAS regained**	**Cumulative incidence [a] % (CI)**	**Crude HR (CI)**	**Adjusted HR [b] (CI)**
**Reoperation**	30 days	Yes	1.5 (1.2–1.7)	Ref.	Ref.
		No	1.6 (1.5–1.8)	1.16 (0.95–1.40)	1.09 (0.89–1.34)
	365 days	Yes	7.5 (7.0–8.0)	Ref.	Ref.
		No	5.8 (5.5–6.2)	0.86 (0.79–0.95)	0.94 (0.85–1.03)

[a] Cumulative incidence for reoperation, treating death as a competing risk.
[b] Adjusted by age, sex, year of surgery, length of hospital stay, and comorbidities.
Abbreviations: CAS: Cumulated Ambulation Score, CI: 95% confidence interval, HR: hazard ratio, Ref.: reference.

Supplementary Table S6. Major reoperation rates by decline in CAS.				
	**Follow-up**	**CAS decline**	**Crude HR (CI)**	**Adjusted HR [a] (CI)**
**Reoperation**	30 days	0 points	Ref.	Ref.
		1-2 points	1.26 (1.00–1.60)	1.20 (0.94–1.52)
		3-4 points	1.11 (0.90–1.38)	1.03 (0.82–1.30)
		5-6 points	0.87 (0.49–1.53)	0.79 (0.44–1.41)
	365 days	0 points	Ref.	Ref.
		1-2 points	0.90 (0.80–1.01)	0.95 (0.84–1.07)
		3-4 points	0.87 (0.78–0.96)	0.95 (0.84–1.06)
		5-6 points	0.57 (0.40–0.79)	0.62 (0.44–0.87)

[a] Adjusted by age, sex, year of surgery, length of hospital stay, and comorbidities.
Abbreviations: CAS: Cumulated Ambulation Score, CI: 95% confidence interval, HR: hazard ratio, Ref.: reference.

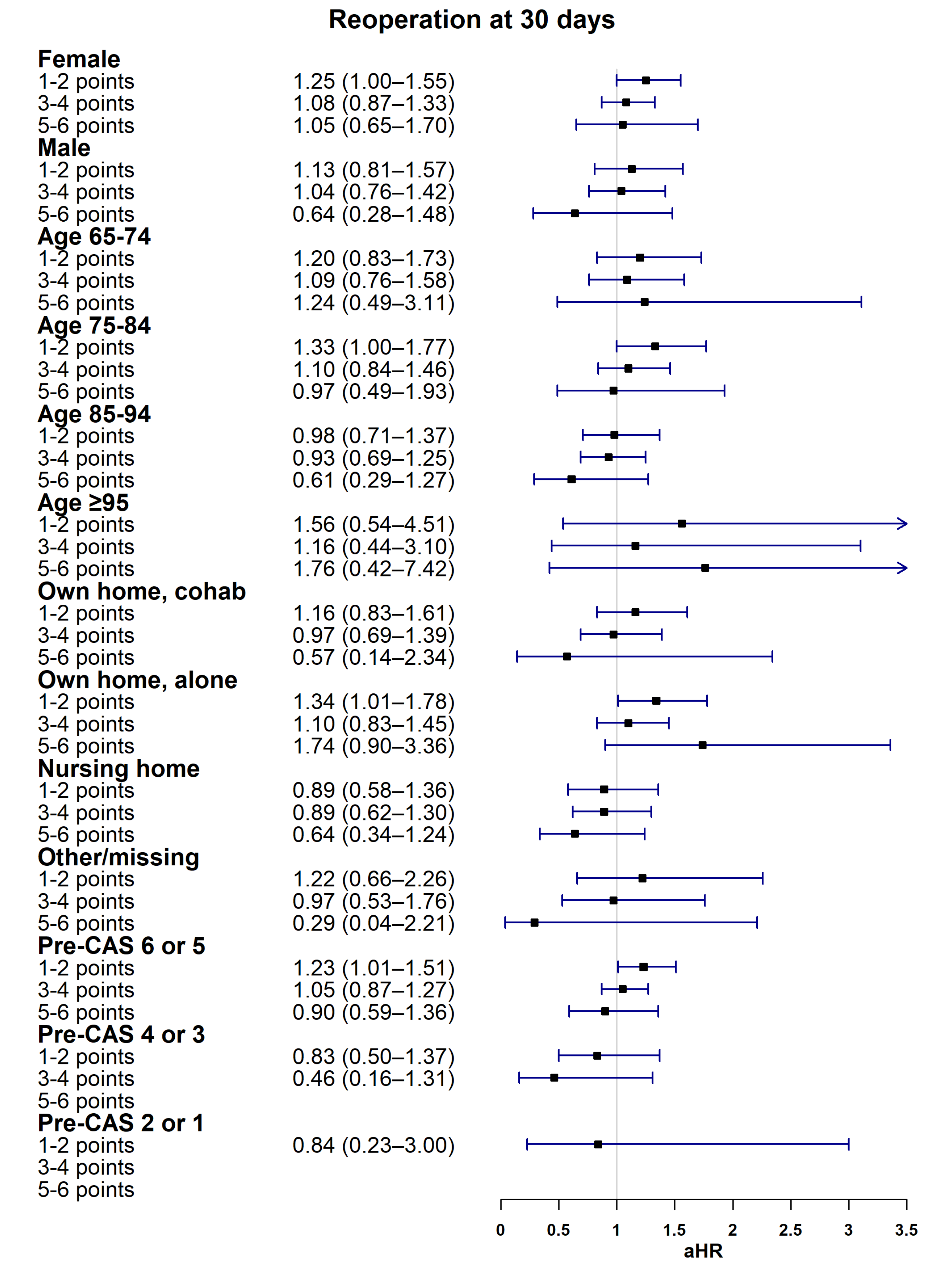

Supplementary Figure S1. Stratified reoperation rates by decline in Cumulated Ambulation Score (CAS) after hip fracture surgery, adjusted by age, sex, year of surgery, length of hospital stay, and comorbidity clusters. AHR: adjusted hazard ratio with 1 (0 CAS points lost) as reference. Cohab: cohabitation.

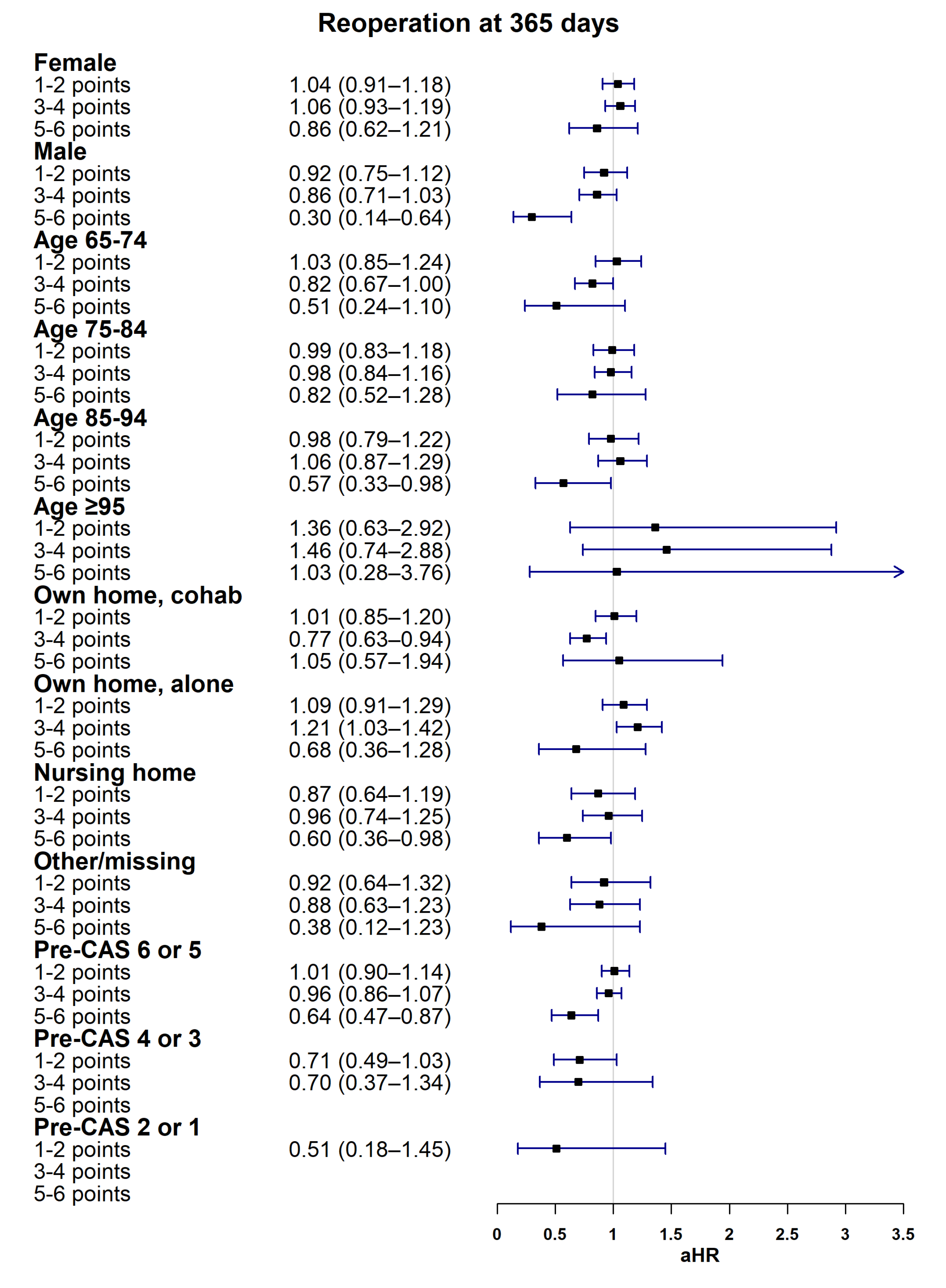

Supplementary Figure S2. Stratified reoperation rates by decline in Cumulated Ambulation Score (CAS) after hip fracture surgery, adjusted by age, sex, year of surgery, length of hospital stay, and comorbidity clusters. aHR: adjusted hazard ratio with 1 (0 CAS points lost) as reference. Cohab: cohabitation.
